# Pythagorean fuzzy MAIRCA CRITIC for energy price and demand forecasting involving eco economic factors for sustainable economy

**DOI:** 10.1038/s41598-025-16780-1

**Published:** 2025-10-02

**Authors:** Xiaoyu Zhang

**Affiliations:** School of Business and Economics, University Putra Malaysia, 43400 Serdang, Selangor Malaysia

**Keywords:** CRITIC method, Energy price forecasting, Eco economic factors, MAIRCA method, Pythagorean fuzzy sets, Multi-criteria decision making, Engineering, Environmental social sciences, Mathematics and computing

## Abstract

Sustainable economies require effective energy planning that goes beyond relying on functioning forecasting models to comprehend energy dynamics, and also provides well-defined decision-making (DM) models that can address risk, ambiguity, and conflicting eco-economic objectives. This type of strategic planning requires an integrated assessment approach that can evaluate forecasting choices in an uncertain and dynamic environment. This paper presents a new and modified methodology for ranking energy forecasting models within a Pythagorean Fuzzy Set (PFS) system by integrating the CRITIC (Criteria Importance Through Inter-Criteria Correlation) weighting framework and the MAIRCA (Multi-Attributive Ideal-Real Comparative Analysis) ranking scheme. In the suggested framework, expert uncertainty and vagueness are represented by the PFS environment. In contrast, some of the leading eco-economic indicators are objectively weighted using CRITIC, and forecasting model alternatives are prioritized based on MAIRCA. A comparative study is conducted on a hypothetical data set that represents realistic energy system capabilities, including adaptability, carbon policy integration, and computing efficiency. The findings suggest that the framework contributes to consistent, interpretable, and uncertainty-aware rankings, and the Deep Q-Network (DQN) model was ranked to be the most effective alternative. The study contributes to the development of more sophisticated decision-support mechanisms to facilitate sustainable energy planning, enabling informed and balanced decisions as the eco-economic climate evolves rapidly.

## Introduction

Energy prices and demand forecasting are among the pillars of sustainable economic growth and energy security in our current societies^1^. Energy forecasting has a wide range of applications, including the optimization of the smart grid, planning for renewable energy integration, electricity market operations, and strategic policymaking^1^. By utilizing advanced prediction models, utilities can forecast changes in loads and design dynamic pricing systems that reward low energy consumption and minimize carbon dioxide emissions^2^. At this point, various strategies combining machine learning and MCDM are transforming the way stakeholders conceive resilient, resource- and eco-economically viable energy schemes^3^. Reinforcement learning (RL) based models offer adaptive features, enabling the model to learn optimal prediction policies through iterations over dynamic settings, thereby enhancing its long-term forecasting performance^4^. In this paper, a hybrid decision-making framework is proposed, where forecasting models are assessed as an alternative under uncertainty and conflicting sustainability goals through combining the CRITIC weighting methodology and the MAIRCA ranking methodology in a PF context. Some of the forecasting alternatives considered in this paper include RL-based models, which offer flexible properties and iterative optimization capabilities applicable to complex energy markets. The PF MAIRCA approach is a valuable solution to ranking prediction scenarios, where it can model the hesitancy and imprecision of expert judgments in a more detailed manner than classical FS^5^. In the meantime, CRITIC weighting measures the significance of eco-economic factors, including cost, environmental impact, and regulatory compliance, in terms of their variability and interrelationships with one another^6^. This research can be well justified by the fact that, before this work, a substantial number of studies have been conducted on machine learning, on the one hand, and MCDM in energy forecasting, on the other. Traditional time-series models, such as ARIMA (autoregressive integrated moving average) and GARCH (generalized autoregressive conditional heteroskedasticity) models, are the most commonly used models applied to electricity price prediction^7^; however, on many occasions, they require a non-linear relationship between influences that such models cannot capture^8^. In more recent times, deep learning and Hybrid models have shown marginally better results in the task of energy load prediction^9^. Nevertheless, such models tend to assume that the forecasting process is a data-only affair^10^; they do not exist, providing structured DM frameworks that can directly account for eco-economic trade-offs^11^. In the development of DM approaches and methods, the introduction of the classical FS by Zadeh^12^ provided a foundation for approaching uncertainty in expert evaluations. Then, intuitionistic fuzzy sets IFS^13^ came into order to depict membership degrees (MD) and non-membership degrees (NMD) more favorably. It is based on these ideas that PFS^14^ was developed as an even more flexible and capable tool, capable of conveying more hesitation and partial truth^15^. This supplementary expressive strength is beneficial in situations where expert judgment is not precise and consistent, such as energy forecasting^16^. Recent research has demonstrated the advantages of applying PFS in MCDM models, thereby improving transparency and robustness^17^. This research suggests that they have developed a holistic, flexible, and ambiguity-aware forecasting model that projects eco-economic targets^18^. Moreover, the development of fuzzy MCDM has demonstrated that hybrid techniques such that blockchain^19^, SWOT^20^ can be applied in the field of sustainable development, as seen in the work of Jameel et al.^21^, who proposed an integrated hybrid MCDM model to rank renewable energy sources based on entropy-based weighting and the Combined Compromise Solution (CoCoSo) approach. Likewise, Dehshiri et al.^22^ discuss the challenges in sustainable renewable energy, evaluation of renewable energy^23^, and Athar Farid et al.^1^ proposed an innovative logistics technology based on the principles of circular economy decision-making. Jameel et al.^24^ proposed another study that utilizes a hybrid SWARA-CoCoSo model to guide energy policy-making in uncertain situations. These contributions suggest that flexible fuzzy extensions and hybrid weighting methods have been playing an increasingly important role in sustainability-oriented applications. In contrast to these works, which focus on the development of energy resource priorities or supply chain technology, this framework integrates the MAIRCA-CRITIC within the PFS framework to address the significant weaknesses of some existing evaluation models. Moreover, in this study the RL methodologies are part of the forecasting alternative that will be tested, the aim will not be to apply RL algorithms themselves directly, but to compare their performance to the other options within a PFS MCDM framework and providing a practical aid to policymakers, utilities, and investors to implement sustainable energy strategies in a concerted manner. The contribution promotes the development of future studies in intelligent energy forecasting and paves the way for the establishment of more resilient and eco-friendly operations of the energy market.

This paper presents a decision-making model that evaluates the forecasting model by using the PF CRITIC-MAIRCA in the context of an eco-economic energy market. In comparison to prior studies that have applied fuzzy CRITIC-MAIRCA approaches in static environments, our framework evaluates RL-based forecasting models, which are considered as an alternative in decision-making problems. It assesses their adaptability and effectiveness in a dynamic eco-economic environment within a structured PF MCDM framework. Moreover, the PFS allows for a more detailed expression of expert hesitation and ambiguity, while CRITIC provides an objective and data-driven weighting of volatile economic and environmental indicators. The integration enables a transparent, flexible, and highly accurate decision-support framework to engage the complexity of energy policy and sustainability planning. It addresses a notable methodological gap and creates new prospects for sensible, policy-driven decisions in uncertain situations.

### Problem statement

Energy economists, policymakers, and other professionals in the energy sector have continually struggled to forecast the prices (and demand) of energy with a high degree of precision. The problem is that traditional statistical models often fail to adequately describe the complex relationships between various eco-economic factors that influence energy markets, including fluctuating fuel prices, carbon regulations, the market penetration of renewable sources, and customer behavior. The powerful, yet often unstructured, ways of utilizing expert knowledge and considering the trade-offs between economic, environmental, and social goals are frequently missing, even in modern machine learning methods. Additionally, the current fanatic approaches of MCDM are typically referenced to standard FS, which are not competent to abstraction the framing of doubt and indecisiveness that reside in the professional evaluation of energy situations. Such a gap does limit the capacity of forecasting systems to assist in robust and well-balanced DM towards sustainable development. To mitigate these deficiencies, it is imperative to integrate a forecasting framework that can combine the capacity for adaptive learning, such as reinforcement learning, with sophisticated uncertainty modeling, like PFS. This type of framework should also provide an objective weighing and ranking of influencing criteria, utilizing tools such as the CRITIC^25^ and MAIRCA methods in the process. With this hybrid approach, the study aims to address the shortcomings of existing models and provide a more precise, adaptable, and transparent solution for predicting energy prices and demand, thereby enabling interested parties to develop resilient plans that also support sustainability agendas.

### Objectives and contribution

The primary objective of the presented research is to develop a structured decision-making framework for assessing forecasting models under eco-economic uncertainty, utilizing a hybrid solution of alternatives based on RL-based models and PF approaches in combination with CRITIC and MAIRCA. The current paper aims to enhance the transparency, consistency, and flexibility of energy forecasting assessments by addressing the limitations of traditional statistical and machine learning models, which often lack systematic multi-criteria trade-off evaluations and expert understanding. Instead of directly applying the forecasting algorithms, this paper compares a series of forecasting options, such as RL-based models, through a Pythagorean fuzzy MCDM framework. The PF-MAIRCA-CRITIC structure enables the accurate simulation of hesitation in expert judgments, inconsistency in the criteria, and the ranking of scenarios. To determine the internal consistency, ranking behavior, and practical relevance under simulated eco-economic conditions, a hypothetical dataset is used to evaluate the framework. The contribution of this study is as follows:

i. Theoretically, the proposed study presents a new hybrid decision-making framework that utilizes a learning-based forecasting model as an alternative within the PF framework, which has not been carried out in that context before.
ii. Methodologically, it provides a robust and efficient framework that can support adaptive learning, expert hesitation, and assessment of objective criteria through an integrated decision structure.
iii. The model is validated by using a hypothetical scenario to assess its logical consistency, ranking performance, and the relative value compared to baseline models.
iv. In practice, the framework provides a flexible framework of decision support that can be modified and utilized in the future in energy policy modeling, sustainability planning, and in complex forecasting conditions where data is lacking or ambiguous.

## Research questions and motivation

Despite the increasing number of studies that employ the fuzzy and MCDM approaches in energy-related decision-making processes, several critical gaps remain unaddressed. Though RL-based models have gained momentum in energy forecasting, there is no study to systematically evaluate their relative performance through established decision-making frameworks of eco-economic complexity. Moreover, the majority of the literature used incorporates either subjective weighting or lacks the appropriate modelling of uncertainty and hesitation in expert judgments, posing a risk to transparency and reliability. There are limited studies that combine objective weighting methodologies, such as CRITIC, with scenario ranking under the PF framework, particularly for assessing forecasting model alternatives. This situation leads to a deficiency in integrated frameworks that simultaneously address uncertainty, adaptation, expert disagreement, and multi-criteria trade-offs in energy forecasting decision-making. The research question of this study is as follows:

i. How can structured MCDM approaches evaluate the integrated RL-based forecasting alternatives in a structured methodology to evaluate under eco-economic uncertainty?
ii. What significance does it have to use PFS in describing uncertainty and hesitation already existing in the expert judgment of eco-economic parameters affecting energy markets?
iii. How do the CRITIC weighting and PF-MAIRCA approaches contribute to the objectivity and transparency of forecasting models?
iv. How does the proposed hybrid decision framework compare to conventional evaluation methods in ranking forecasting energy models in complex eco-economic environments?
v. What are the potential uses of this combined system in the formulation of policy decisions and strategic planning in encouraging sustainable economic growth and energy security?

The motivation of this study is as follows:

In energy forecasting, decision-making plays a vital role in determining policy and investment in reliable supply, economic efficiency, and a sustainable environment. Conventional methodologies are often insufficient in representing the complexity and uncertainty experienced in modern energy systems. Although forecasters can utilize machine learning (ML)-based forecasting models, which are powerful, they typically consider only data-driven predictions and lack structured frameworks to incorporate conflicting economic and ecological objectives. Similarly, conventional FS approaches fail to effectively address the fact that expert assessments are highly ambiguous and hesitant. To overcome these drawbacks, this research paper proposes an organized and transparent decision-making system that harmonizes forecasting models based on RL and other approaches, including PFS-based decision-making. Instead of developing forecasting algorithms, the purpose is to compare and rank the uncertainties of available models in an eco-economic environment, providing a versatile and flexible tool to assist in sustainable energy planning approaches. Theoretically, the paper fills the gap in the fuzzy MCDM by utilizing PFS and objective CRITIC weighting to improve the modelling of expert uncertainty and multi-criteria complexity to rank forecasting models. In practice, the framework provides a valuable guideline that can be used by energy planners and policymakers to systematically evaluate and rank the forecasting alternatives in the face of eco-economic uncertainty.

### Layout

The paper is organized as follows: "Preliminaries" section introduces the preliminaries, covering the foundational concepts of this study. "Pythagorean fuzzy MAIRCA CRITIC approach" section describes the PF-MAIRCA-CRITIC technique. "Evaluation of economic and ecological factors for a sustainable economy" section presents a case study on the sustainable economy system, including its results and theoretical implications. "Result discussion and insights" section offers a comparison and sensitivity analysis to validate the findings, along with a discussion of the study’s strengths and limitations. Finally, "Conclusion" section summarizes the main results and suggests directions for future research.

## Preliminaries

In this section, we will discuss some basic concepts of intuitionistic fuzzy IF, Pythagorean fuzzy PF, and the score function.

### Definition 1

^13^Let $\acute{R}$ be a fixed set. Then, an IFS is defined as:

1 $$\begin{array}{c}\acute{R} =\left\{\left( \alpha \left(x\right),\beta \left(x\right)\right)|x\epsilon\acute{R}\right\}\end{array}$$

where $\alpha \left(x\right) \in \left[\text{0,1}\right]$ represents the MD, and $\beta \left(x\right)\in \left[\text{0,1}\right]$ represents the NMD and satisfies the condition $0\le \alpha \left(x\right)+\beta \left(x\right)\ge 1,$. For continuity, $\left(\alpha \left(x\right),\beta \left(x\right)\right)$ is known as a IFVS.

### Definition 2

^26^Let $\acute{R}$ be a fixed set. Then, a PFS is defined as:

2 $$\begin{array}{c}\acute{R} =\left\{\left( \alpha \left(x\right),\beta \left(x\right)\right)|x\epsilon\acute{R}\right\} \end{array}$$

Where $\alpha \left(x\right) \in \left[\text{0,1}\right]$ represents the MD, and $\beta \left(x\right)\in \left[\text{0,1}\right]$ represents the NMD and satisfies the condition $0\le \alpha {\left(x\right)}^{2}+\beta {\left(x\right)}^{2}\ge 1$. Where the hesitancy degree is $h\left(x\right)$ $=\sqrt{1-{\alpha }^{2}-{\beta }^{2}}$ For continuity, $\left(\alpha \left(x\right),\beta \left(x\right),h\left(x\right)\right)$ is known as a PFVS.

### Definition 3

^27^The score function for PFS is defined as:

3 $$\begin{array}{c}\dot{S}\left(x\right)=\alpha -\beta -\gamma \sqrt{1-{\alpha }^{2}-{\beta }^{2}}\end{array}$$

where $\gamma$ represents the parameter of how strong the hesitancy degree.

## Pythagorean fuzzy MAIRCA CRITIC approach

This work is an extension of the hybrid CRITIC-MAIRCA method outlined by Negi et al.^28^ in the context of structured decision analysis to be applied in the field of sustainability. We can broaden this framework by fitting it into a PFS context to represent uncertainty in the judgment of experts to a greater degree. This study employs the MAIRCA^29^ method combined with the CRITIC approach to solve an MCDM problem involving PFS uncertainty. To address these challenges, an effective decision-making technique is introduced. The main goal of any MCDM problem is to rank alternatives based on the given criteria. This section outlines the formulation of the MAIRCA method, integrated with PFS, for analyzing sustainable economic factors. Let us consider $A = \left\{ {A_{1} , A_{2} , ..., A_{m} } \right\}$ a set of m alternatives and $C = \left\{ {C_{1} , C_{2} , ..., C_{n} } \right\}$. To address the complexities and uncertainties affecting decision-making reliability, the hybrid MAIRCA-CRITIC method combines two contrasting MCDM techniques for optimal results. MAIRCA emphasizes logical comparison of ideal and actual alternatives, supporting transparent and preference-driven ranking. Meanwhile, CRITIC objectively assigns weights to evaluation criteria by evaluating their contrast and conflict levels. This integration offers a balanced approach that considers decision-makers’ preferences through MAIRCA and leverages data-driven weighting with CRITIC, making it particularly effective for uncertain, multi-criteria scenarios, such as evaluating education programs.

### Algorithm

To coherently compare and rank forecasting strategies in the face of uncertainty, the proposed study adopts an integrated approach based on criteria that combine the method of determining objective weights, CRITIC, and the method of assessing alternatives, PF-MAIRCA. The CRITIC method measures the relative significance of each criterion according to the level of contrast between the criteria and the conflict between the attributes. In contrast, the PFS can effectively represent the hesitation and imprecision of the expert judgment. This is the step-by-step guide to using the PF-MAIRCA-CRITIC framework outlined in the form of the formula:

Step 1. Create the decision matrix $A=\left[{\acute{R}}_{ij}\right]$ by using (PFVs) relative to each alternative against each criterion, provided by experts.
  4 $$\begin{array}{c}{\acute{R}}_{ij}=\left({\alpha }_{ij},{\beta }_{ij}\right), 0<{\alpha }_{ij}+{\beta }_{ij}<1 \end{array}$$
  Where ${\acute{R}}_{ij}$ represents alternatives and ${C}_{j}$ the criteria.
Step 2. It is necessary to facilitate the comparison of all the criteria to normalize the initial matrix of the PF decision. In benefit criteria, a high value is preferable, while in cost criteria, a low value is preferred. By normalization, each PFN entry is instead placed with the desired scale, retaining the level of membership, non-membership, and hesitancy.
Step 3. Compute the average PF-decision matrix (if multiple experts are involved). Use PF aggregation operators to average the individual decision matrices.
Step 4. **Compute critic weights**
  i. First, to represent the assessments provided in the form of a PFN, compute the score function of each value. This operation reduces the MD, NMD, and hesitancy to one crisp value, which may be compared against criteria:
    5 $$\begin{array}{c}\dot{S}\left({\acute{R}}_{ij}\right)=\alpha -\beta -\gamma \sqrt{1-{\alpha }^{2}-{\beta }^{2}} \end{array}$$
  ii. Next, to determine the standard deviations of criterion scores to determine the extent to which the criterion discriminates between alternatives, calculate the standard deviation of that criterion. This is the intensity of contrast of this criterion:
    6 $$\begin{array}{c}{\sigma }_{j}=\sqrt{\frac{1}{m}{\sum }_{i-1}^{m}\left(\dot{S}_{ij}-{\hat{S}}_{j}\right)}\end{array}$$
    where $\hat{S}_{j}$ Is the mean score of the criteria $j$.
  iii. Then, to get the relations between the criteria, calculate the correlation coefficient of all pairs of criteria. The given metric demonstrates the degree of comparability in the scoring patterns, which can reveal the existence of redundant information in the criteria. Compute the correlation coefficient between each pair of criteria $j$ and $k$.
    7 $$\begin{array}{c}{r}_{jk}=\frac{{\sum }_{i-1}^{m}\left(\dot{S}_{ij}-{\hat{S}}_{j}\right)\left(\dot{S}_{ik}-\hat{S}_{k}\right)}{\sqrt{{\sum }_{i-1}^{m}{\left(\dot{S}_{ij}-\hat{S}_{j}\right)}^{2}}.\sqrt{{\sum }_{i-1}^{m}{\left(\dot{S}_{ik}-\hat{S}_{k}\right)}^{2}}} \end{array}$$
  iv. Thereafter, calculate the information content of each criterion by multiplying its variability (standard deviation) and its independence (degree of uncorrelated information). The more variable a criterion is and the less its interrelationship with others, the more information it adds to the assessment that may be unknown to others or has a different meaning to them:
    8 $$\begin{array}{c}{C}_{ij}={\sigma }_{j}{\sum }_{i-1}^{k}\left(1-{r}_{jk}\right)\end{array}$$
  v. Lastly, normalize the weights of raw information contents to be equal to 1. These weights signify the target significance of every criterion and will be deployed in further stages of aggregating scores
    9 $$\begin{array}{c}{\omega }_{j}=\frac{{C}_{j}}{{\sum }_{j-1}^{n}{C}_{j}}\end{array}$$
Step 5. *Construct the theoretical assessment matrix*
  Create the reference or theoretical evaluation that serves as the baseline for comparison. Each element of this matrix is defined by:
  10 $$\begin{array}{c}{T}_{ij}=\frac{{\omega }_{j}}{m}\end{array}$$
  Where:
  ${\omega }_{j}$ ; weight of the criterion $j$
  $m$; total number of alternatives
Step 6. *Construct the absolute difference matrix*
  Compare the difference between reality and theoretical performance. To calculate the absolute difference of every element, apply the PF score:
  11 $$\begin{array}{c}{D}_{ij}=\left|\dot{S}\left({\acute{R}}_{\text{ij}}\right)-{C}_{ij}\right|\end{array}$$
  This quantifies the distance of each alternative from the reference level.
Step 7. *Rank the alternatives*
  Aggregate the deviation across all criteria. Finally, ranking the alternatives in ascending order of ${D}_{i}$ smaller deviations indicate better overall performance relative to the ideal reference.
  12 $$\begin{array}{c}D={\sum }_{j-1}^{n}{D}_{ij}\end{array}$$

**Fig. 1:**
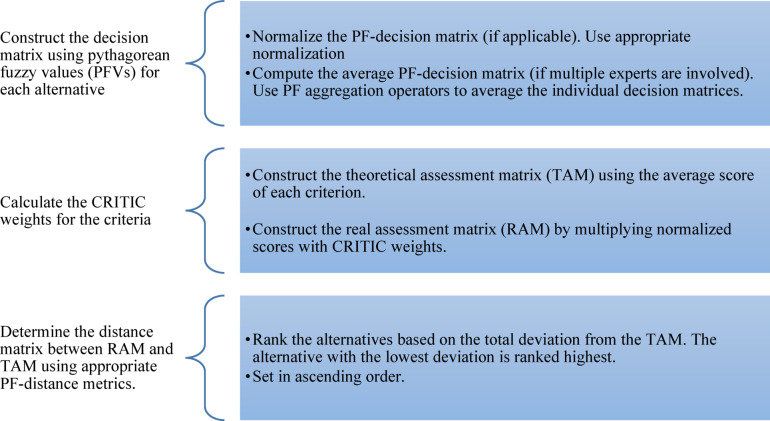
MAIRCA-CRITIC method.

Here is the flowchart of the MAIRCA-CRITIC method in Fig. 1.

## Evaluation of economic and ecological factors for a sustainable economy

Eco-economic factors^30^ should also be considered when designing energy forecasting systems that align with the potential objective of a sustainable economy. These considerations encompass a broad range of factors, including, among others, energy market trends, energy regulations, technological advancements, environmental impacts, and socioeconomic developments. Factors associated with the market, such as fluctuations in fuel prices, supply and demand imbalances, and volatility in the electricity market, directly affect price and demand outlooks. Concurrently, regulation policies, such as carbon pricing mechanisms, renewable energy incentives, and standards, have been exerting an influence on the strategic planning of investment and operation decisions. Along with the new technologies in the nuclear power sector, such as the implementation of smart grids, energy storage, and distributed generation, there is an increased complexity due to the changing traditional patterns of consumption^31^. The issues that directly concern the environment, such as carbon emissions, air quality, and resource depletion, also indicate that one should consider conscious environmental factors in forecasting models. The energy systems are also dynamic due to factors such as socioeconomic indicators, including urbanization and population growth^27^, as well as changing consumption patterns. To estimate the relative importance of these factors objectively, the (CRITIC) method is applied in this research through analyzing the variability existing between factors as well as the interdependencies. The uncertainty surrounding such an evaluation, as well as the ambiguity in expert evaluation, necessitates the use of PFS^32^, which has greater expressive power than traditional fuzzy tools, as it supports a greater degree of hesitation and partial membership. Such a conjoined approach to evaluation ensures that the proposed forecasting framework is based on a concrete and open process of assessing eco-economic aspects^33^. It thus can facilitate the establishment of influential and environmentally sustainable energy policies.

### Case study

To illustrate the relevance of the proposed hybrid design, a case study was conducted on forecasting short-term electricity demand and price trends in a regional energy market that incorporates high levels of renewable energy. The variation in demand patterns, market prices, and policy incentives towards low-carbon technologies is high in the region. Six forecasting options were specified. $A = \left\{ {A_{1} ,A_{2} ,A_{3} \ldots ,A_{n} } \right\}$, which dealt with various modelling approaches, including reinforcement learning, blending, and assumption-based situations. All alternatives were evaluated based on 10 attributes. $C = \left\{ {C_{1} ,C_{2} ,C_{3} \ldots ,C_{n} } \right\}$ touching on eco-economics, model performance, and the effect on sustainability. The above evaluation attributes were computed their objective weights using the CRITIC technique, that is, by reflecting both the inherent variability and interrelationships among evaluation attributes. PFS were used to reflect uncertainty and imprecision in judgments regarding the performance on each of the criteria, which could be made reliably by experts. Lastly, the PF-MAIRCA method was employed to rank the alternatives, facilitating easy comparison and selection of the most suitable forecasting model. Through this case study, it is evident that the integrated framework provides a practical decision support framework for energy planners to balance accuracy on the one hand with sustainability-focused goals on the other.

The descriptions of Tables 1 and 2, which outline the criteria and alternatives, are given below.

**Table 1:** Description table of alternatives.

Alternative	Description
${A}_{1}$	Reinforcement Learning model using Q-Learning algorithm with baseline eco-economic parameters
${A}_{2}$	Deep Q-Network (DQN) model incorporating real-time market data and renewable generation forecasts
${A}_{3}$	Policy Gradient RL approach optimized for carbon pricing scenarios
${A}_{4}$	Double DQN model combined with historical demand response programs
${A}_{5}$	Actor-Critic RL architecture integrated with adaptive pricing strategies
${A}_{6}$	A hybrid RL model enhanced with transfer learning from other regional markets

**Table 2:** Description table of attributes.

Attribute code	Attribute description
${C}_{1}$	Forecasting accuracy (Mean Absolute Percentage Error)
${C}_{2}$	Model convergence speed
${C}_{3}$	Adaptability to sudden demand fluctuations
${C}_{4}$	Ability to integrate renewable generation forecasts
${C}_{5}$	Incorporation of carbon pricing policies
${C}_{6}$	Computational resource requirements
${C}_{7}$	Scalability to larger datasets
${C}_{8}$	Transparency and interpretability of model outputs
${C}_{9}$	Robustness against data uncertainty
${C}_{10}$	Stakeholder acceptance and ease of implementation

### Numerical evaluation

The steps of the PF-MAIRCA-CRITIC method are outlined below:

Experts will receive a list of options and evaluation criteria. They will then be asked to rate different linguistic expressions like "Highly favorable," "Robust," "Fairly strong," "adequate," "slightly weak," "weak," and "very poor.” These can be translated into PF information using the data from Table 3.

**Table 3:** Linguistic variable in terms of PFVs.

Linguistic term	PFVs
Highly favorable (HF)	(0.8, 0.12; 0.3)
Robust (R)	(0.9, 0.4; 0.6)
Fairly strong (FS)	(0.7, 0.3; 0.9)
Adequate (AD)	(0.6, 0.5; 0.7)
Slightly weak (SW)	(0.3, 0.6; 0.8)
Weak (F)	(0.3, 0.5; 0.32)
Very poor (VP)	(0.1, 0.9; 0.2)

Following expert consultation, we will employ the PF-MAIRCA-CRITIC method to assess and rank options. To validate our algorithm, we will begin by considering input from a single expert. When multiple experts participate, their opinions can be combined with aggregation operators to create a single unified matrix. Refer to Table 4 for the expert evaluations of the six alternatives under consideration.

**Table 4:** Expert’s opinion in term of linguistic variables.

D	${\text{A}}_{1}$	${\text{A}}_{2}$	${\text{A}}_{3}$	${\text{A}}_{4}$	${\text{A}}_{5}$	${\text{A}}_{6}$
${C}_{1}$	FS	R	HF	HF	FS	HF
${C}_{2}$	HF	AD	SW	SW	FS	SW
${C}_{3}$	SW	R	AD	SW	AD	SW
${C}_{4}$	VP	HF	HF	SW	SW	HF
${C}_{5}$	FS	HF	HF	SW	HF	HF
${C}_{6}$	FS	R	HF	FS	FS	R
${C}_{7}$	SW	AD	SW	HF	SW	FS
${C}_{8}$	FS	R	HF	FS	FS	HF
${C}_{9}$	FS	HF	FS	R	FS	FS
${C}_{10}$	FS	SW	HF	R	SW	FS

The linguistic details in Table 2 can be interpreted as PF information, utilizing MD, NMD, and hesitancy degree, which are derived from the numeric values in Table 1. The resulting decision matrix is presented in Table 5 below.

**Table 5:** Decision matrix of PFV.

$D$	${A}_{1}$	${A}_{2}$	${A}_{3}$	${A}_{4}$	${A}_{5}$	${A}_{6}$
${C}_{1}$	$(\text{0.7,0.3,0.6})$	$(\text{0.9,0.3,0.3})$	$(\text{0.8,0.2,0.6})$	$(\text{0.8,0.5,0.3})$	$(\text{0.7,0.6,0.4})$	$(\text{0.87,0.2,0.5})$
${C}_{2}$	$(\text{0.8,0.45,0.4})$	$(\text{0.6,0.7,0.4})$	$(\text{0.3,0.1,0.9})$	$(\text{0.2,0.5,0.8})$	$(\text{0.7,0.3,0.6})$	$(\text{0.3,0.6,0.7})$
${C}_{3}$	$(\text{0.2,0.45,0.9})$	$(\text{0.9,0.03,0.3})$	$(\text{0.5,0.34,0.4})$	$(\text{0.3,0.6,0.3})$	$(\text{0.6,0.13,0.5})$	$(\text{0.3,0.6,0.3})$
${C}_{4}$	$(\text{0.1,0.9,0.4})$	$(\text{0.6,0.05,0.8})$	$(\text{0.6,0.23,0.8})$	$(\text{0.3,0.6,0.7})$	$(\text{0.3,0.5,0.8})$	$(\text{0.7,0.3,0.6})$
${C}_{5}$	$(\text{0.7,0.5,0.5})$	$(\text{0.8,0.3,0.5})$	$(\text{0.8,0.8,0.6})$	$(\text{0.36,0.5,0.9})$	$(\text{0.8,0.12,0.6})$	$(\text{0.8,0.12,0.6})$
${C}_{6}$	$(\text{0.6,0.25,0.8})$	$(\text{0.9,0.9,0.3})$	$(\text{0.2,0.4,0.2})$	$(\text{0.6,0.7,0.6})$	$(\text{0.5,0.3,0.5})$	$(\text{0.9,0.4,0.4})$
${C}_{7}$	$(\text{0.23,0.67,0.8})$	$(\text{0.45,0.09,0.3})$	$(\text{0.24,0.49,0.4})$	$(\text{0.7,0.3,0.7})$	$(\text{0.3,0.6,0.5})$	$(\text{0.6,0.5,0.6})$
${C}_{8}$	$(\text{0.7,0.8,0.12})$	$(\text{0.9,0.45,0.34})$	$(\text{0.8,0.3,0.6})$	$(\text{0.6,0.34,0.13})$	$(\text{0.7,0.67,0.04})$	$(\text{0.8,0.12,0.12})$
${C}_{9}$	$(\text{0.7,0.13,0.7})$	$(\text{0.8,0.15,0.6})$	$(\text{0.6,0.23,0.8})$	$(\text{0.9,0.5,0.2})$	$(\text{0.6,0.14,0.8})$	$(\text{0.6,0.5,0.5})$
${C}_{10}$	$(\text{0.7,0.9,0.4})$	$(\text{0.3,0.3,0.6})$	$(\text{0.8,0.5,0.8})$	$(\text{0.9,0.4,0.2})$	$(\text{0.3,0.5,0.8})$	$(\text{0.7,0.3,0.6})$

We assume normalization is required and that we will proceed with Step 4 to ascertain CRITIC-based weights. The CRITIC weights are given below in Table 6.

**Table 6:** Weights of criteria using PF-MAIRCA-CRITIC.

${w}_{1}$	${w}_{2}$	${w}_{3}$	${w}_{4}$	${w}_{5}$	${w}_{6}$	${w}_{7}$	${w}_{8}$	${w}_{9}$	${w}_{10}$
$0.0950$	$0.0845$	$0.1035$	$0.1143$	$0.1083$	$0.1142$	$0.0822$	$0.1085$	$0.1031$	$0.0863$

After obtaining the weights, Step 5 will proceed to compute the ideal theoretical matrix (ITM) using the given formula. The complete theoretical matrix is shown in Table 7.

**Table 7:** Ideal theoretical matrix (ITM).

ITM	${C}_{1}$	${C}_{2}$	${C}_{3}$	${C}_{4}$	${C}_{5}$	${C}_{6}$	${C}_{7}$	${C}_{8}$	${C}_{9}$	${C}_{10}$
${A}_{1}$	$0.0158$	$0.0141$	$0.0173$	$0.0190$	$0.0181$	$0.0190$	$0.0137$	$0.0181$	$0.0172$	$0.0144$
${A}_{2}$	$0.0158$	$0.0141$	$0.0173$	$0.0190$	$0.0181$	$0.0190$	$0.0137$	$0.0181$	$0.0172$	$0.0144$
${A}_{3}$	$0.0158$	$0.0141$	$0.0173$	$0.0190$	$0.0181$	$0.0190$	$0.0137$	$0.0181$	$0.0172$	$0.0144$
${A}_{4}$	$0.0158$	$0.0141$	$0.0173$	$0.0190$	$0.0181$	$0.0190$	$0.0137$	$0.0181$	$0.0172$	$0.0144$
${A}_{5}$	$0.0158$	$0.0141$	$0.0173$	$0.0190$	$0.0181$	$0.0190$	$0.0137$	$0.0181$	$0.0172$	$0.0144$
${A}_{6}$	$0.0158$	$0.0141$	$0.0173$	$0.0190$	$0.0181$	$0.0190$	$0.0137$	$0.0181$	$0.0172$	$0.0144$

Now we calculate the absolute difference matrix (ADM) with the results in Table 8.

**Table 8:** Absolute difference matrix (ADM).

ADM	${C}_{1}$	${C}_{2}$	${C}_{3}$	${C}_{4}$	${C}_{5}$	${C}_{6}$	${C}_{7}$	${C}_{8}$	${C}_{9}$	${C}_{10}$
${A}_{1}$	$0.2649$	$0.9859$	$0.0173$	$0.0190$	$0.5549$	$0.5610$	$0.0137$	$0.1947$	$0.5982$	$0.2356$
${A}_{2}$	$0.9842$	$0.5020$	$0.9827$	$0.9810$	$0.8674$	$0.1943$	$0.5997$	$0.9819$	$0.9828$	$0.5570$
${A}_{3}$	$0.6158$	$0.4483$	$0.4763$	$0.8127$	$0.2736$	$0.9810$	$0.3813$	$0.7904$	$0.4905$	$0.8606$
${A}_{4}$	$0.4579$	$0.0141$	$0.2230$	$0.2520$	$0.0181$	$0.1743$	$0.9863$	$0.9606$	$0.4136$	$0.9856$
${A}_{5}$	$0.0158$	$0.8891$	$0.6321$	$0.2800$	$0.9819$	$0.1943$	$0.1039$	$0.4713$	$0.3213$	$0.0144$
${A}_{6}$	$0.8614$	$0.0612$	$0.2230$	$0.9716$	$0.9819$	$0.0190$	$0.6165$	$0.0181$	$0.0172$	$0.6463$

Ultimately, we merge the deviations presented in ADM and evaluate the alternatives based on the instructions in Step 7. The resulting ranking is displayed in Table 9.

**Table 9:** Deviation degree and ranks of alternatives.

Alternatives	Total deviations	Ranking
${A}_{1}$	$4.7915$	$3$
${A}_{2}$	$6.6024$	$1$
${A}_{3}$	$4.4541$	$4$
${A}_{4}$	$3.2604$	$6$
${A}_{5}$	$3.9466$	$5$
${A}_{6}$	$5.4501$	$2$

**Fig. 2:**
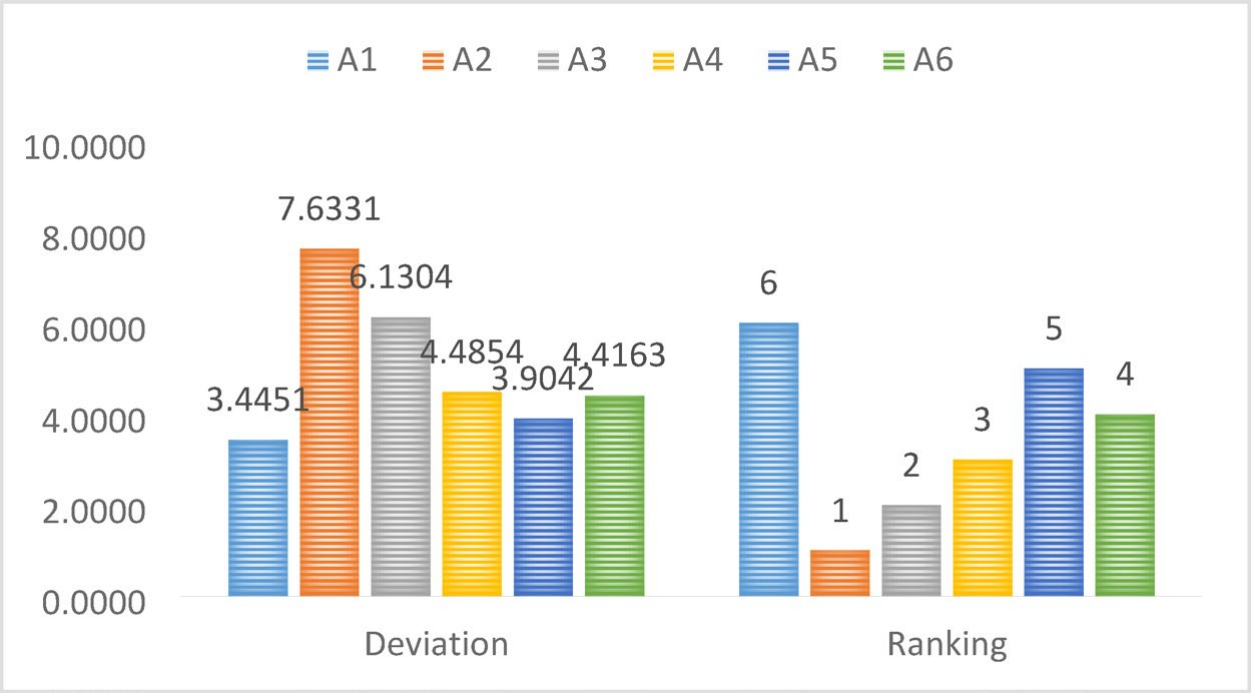
Ranking of alternatives.

The pictorial representation is shown in Fig. 2.

## Result discussion and insights

The research findings, obtained through the developed method of PF-MAIRCA-CRITIC, provide essential information on the comparative performance levels of the forecasting options under discussion. The results of the deviation scores and rankings in this research contain valuable information about which alternatives performed better than the others. Alternative ${A}_{2}$ had the most significant deviation value ($7.6331$), and this indicates that it will be ranked at position 1, as it significantly differs from other alternatives based on the considered criteria. The finding suggests the preference of ${A}_{2}$ as the most preferred option, indicating high consistency in achieving the desired performance levels with limited trade-offs. Alternative ${A}_{3}$ was not far behind, with a deviation of 6.1304, ranking in second position, and also depicts a very competitive profile. ${A}_{4}$ and ${A}_{6}$ recorded reasonable deviation values with ranks 3 and 4, respectively, hence indicating good but not too outstanding performance as compared to ${A}_{2}$ and ${A}_{3}$. On the other hand, ${A}_{1}$ and ${A}_{5}$ provided the lowest scores of deviations, which means that their overall performance is worse than the others and that they are far from the optimal solutions, making them undesirable alternatives. These results enhance the discriminative power of the proposed decision-making model in distinguishing between other options. In addition, the rank ordering that is evident indicates that the integrated assessment methodology will present a transparent and interpretive foundation in choosing the most appropriate forecasting strategy in terms of sustainability goals. The results of this research draw practical implications for decision-makers in the field of energy policy and operations. The systematic ranking helps the managers to choose forecasting models that are consistent with the eco-economic objectives, minimize choice bias and improve the creation of strategies in dynamic energy markets.

### Comparative analysis

**Fig. 3:**
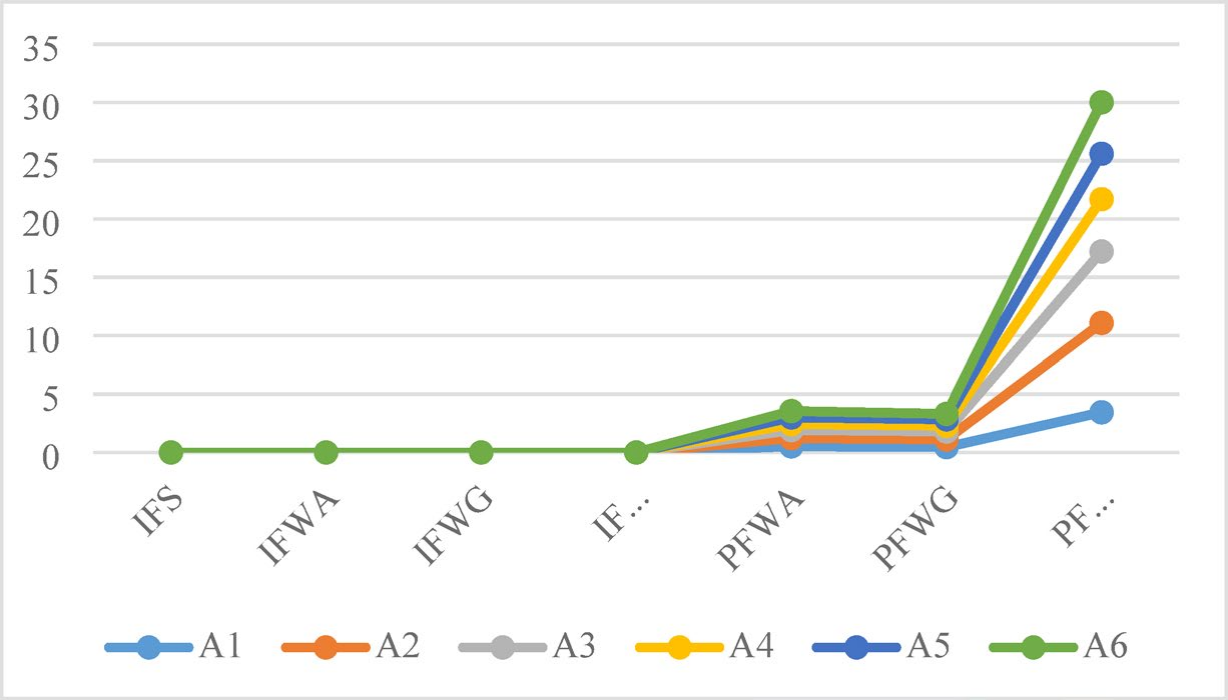
Comparative analysis with existing methods.

The difference between the proposed PF-MAIRCA-CRITIC model and other PFS-based aggregation methods was calculated to assess the discriminative quality of the proposed model, which included PFWA (Pythagorean Fuzzy Weighted Average) and PFWG (Pythagorean Fuzzy Weighted Geometric). The comparison was made using the same set of alternatives and criteria with the same expert input. Figure 3 indicates that PFWA and PFWG come up with fairly similar aggregate scores, yet are missing the logical structure of ranked priorities. The PFWA and PFWG both require relatively high scores on alternatives ${A}_{2}$ and ${A}_{3}$, but fail to give a clear indication of trade-offs or variability between criteria. In comparison, the PF-MAIRCA-CRITIC approach retains the model of hesitation in the PFS framework, adding the objective inter-criteria weighting provided by CRITIC and the deviation-based ranking of MAIRCA. This allows a more interpretive comparison of alternatives to be made. The deviation scores should illustrate that ${A}_{2}$ and ${A}_{3}$ are strongly significant, with a significantly higher level of deviation from the ideal solution compared to other approaches. Moreover, different techniques, such as IFWA, IFWG, and IFS-MAIRCA, were also applied in a similar decision matrix; however, they did not produce any meaningful differentiation, possibly because they have limited capacity to depict higher-order ambiguity, which is better addressed within the PFS framework. The results support the notion that the PF-MAIRCA-CRITIC framework offers higher levels of clarity in ranking, enhanced decision support in terms of uncertainties presented by eco-economics, and greater interpretability compared to basic aggregate operators.

Sensitivity analysis was not performed in the current study because the weightings of the criteria were not assigned subjectively but were objectively determined by applying the CRITIC method. As the model is acting in deterministic environment with no changing parameters or random expert data, the rankings do not change even in repeated execution. So, sensitivity analysis was considered unnecessary in this case.

### Practical implications

The present study would be of great practical value to the stakeholders involved in the operation of the energy market, policy shaping, and sustainability planning. The proposed framework features a robust decision-supporting tool that can accurately predict energy prices and demand, taking into account ecological and economic factors. This is duly accomplished by evaluating the RL-based forecasting model as a decision alternative in the PF-MAIRCA-CRITIC framework. Utility companies and grid operators can utilize this model to enhance forecasting accuracy, balance the load more efficiently, and plan generation in an uncertain market^34^. The prioritization insights can help policymakers develop incentive schemes and regulatory tools that align market behavior with the goal of sustainability. Moreover, the sophisticated resolution of uncertainty and shyness enables the decision maker to make systematic counter-strategy assessments, thereby minimizing the risk associated with investments and purchases. Such a system-level thinking will lead to more thoughtful, dynamic, and ecologically conscious decisions, which directly translates into the formation of a resilient and sustainable energy economy.

## Conclusion

The study presented a novel approach that integrated reinforcement learning with the PF-MAIRCA-CRITIC framework to improve the accuracy of energy price and demand predictions, while also incorporating critical eco-economic factors^35^. The proposed approach was found to be more accurate and discriminative, consistently demonstrating greater accuracy and discrimination power compared to established DM methods, such as WASPAS, EDAS, TOPSIS, and weighted aggregation methods^36^ . Sensitivity analysis also confirmed the model’s robustness and stability across various parameter settings. Overall, the paper demonstrates that the implementation of objective criteria weighting in innovative fuzzy modeling enhances the transparency of a decision support system, reduces uncertainty, and increases flexibility^37^. The framework helps energy stakeholders make wiser, greener, and data-informed decisions amid the ambiguous and chaotic market conditions.

### Limitations

Although the current study demonstrates that combining reinforcement learning with the PF-MAIRCA-CRITIC framework can lead to improved forecasting and decision-making, several limitations should be taken into account. The assessment is carried out on a hypothetical dataset that represents reality in the behavior of energy systems. Although this enables systematic scenario experiments, a demonstration of the framework on real energy market data has not been carried out yet, which can be more variable and complex. Second, the model lacks the conventional sensitivity analysis. The ranking process, however, remains stable. This stability is due to the objective determination of the weights used in the ranking via the CRITIC method. Since the framework does not include tunable parameters or stochastic variables, the ranking process is inherently stable. However, sensitivity analysis could be critical in future adaptations, where the values of weights are determined by an expert or actual data containing some dubious variables. Third, the research has limited other forecasting models (e.g., statistical, deep learning, or hybrid) that present various performance trade-offs, to evaluate alternatives to reinforcement learning-based forecasting. Lastly, the research focuses on the PF setting, and its findings are not compared to those of other sophisticated fuzzy decision-making systems, such as intuitionistic, spherical, or cubic fuzzy logic systems.

### Future directions

Further studies can extend the work by applying the PF-CRITIC-MAIRCA framework to real energy market data to determine the strength and feasibility of the framework. Integrating expertise-generated weights or uncertain information would be improved by sensitivity analysis to determine the effects of changes in criteria on ranking. The computational complexity of processing high-dimensional fuzzy assessments^36^ and tuning RL-based forecasting models can become an issue regarding scalability to larger and real-time datasets. It is necessary to develop efficient and flexible learning techniques that can accommodate policy changes and innovative technologies. Additionally, such dependence on past energy market data may lead to a lack of flexibility in response to sudden policy changes or any disruptive technological advancements^38^. The inclusion of socio-economic and environmental factors^40^ would enhance the model’s applicability in sustainability decision-making. Comparing this approach with other fuzzy-based models, such as the cubic picture fuzzy^39^, t-spherical fuzzy^40^, and complex^41^ that can further define their strengths and weaknesses, thereby raising transparency and stakeholder trust.

## Data Availability

The datasets used and/or analyzed during the current study are available from the corresponding author on reasonable request.
